# Fibroepithelioma of pinkus in a 9-year-old boy: a case report

**DOI:** 10.1186/1757-1626-1-21

**Published:** 2008-06-27

**Authors:** Zenggang Pan, Nhi Huynh, Deba P Sarma

**Affiliations:** 1 Department of Pathology, Creighton University Medical Center, Omaha, NE 68131, USA

## Abstract

Fibroepithelioma of Pinkus (FEP) is a rare indolent variety of basal cell carcinoma that is typically polypoid and located on the trunk of adult males aged 40–60 years. Basal cell carcinoma (including FEP) is very rare in the pediatric population. We are reporting such a case occurring in a 9-year-old boy.

## Case presentation

**Figure 1:**
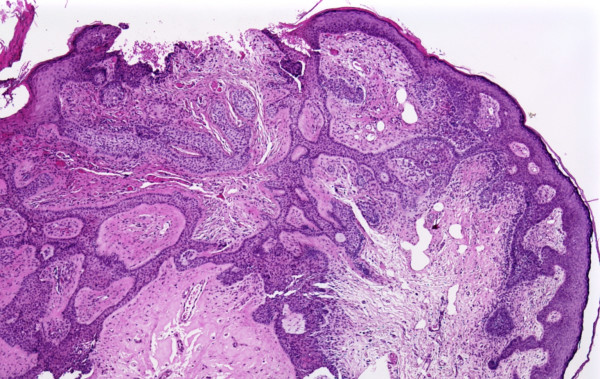


A 9-year-old boy presented with a 6.0-mm polypoid erythematous nodule with ulceration on his left chest. An excisional biopsy was done. Histologically, the tumor revealed several foci of superficial basal cell carcinoma along the epidermis. In the dermis, the tumor was composed of basaloid epithelial anastomosing cords that were separated by fibrovascular stroma connected to the overlying epidermis (Figure 1). The histopathological features were that of a fibroepithelioma of Pinkus. The lesion was completely removed with clear biopsy margin. The patient is being followed for any possible local recurrence.

## Discussion

Fibroepithelioma of Pinkus (FEP) was first described as *premalignant fibroepithelial tumor of the skin *by Herman Pinkus in 1953 [1]. Although FEP is currently accepted as a variant of basal cell carcinoma (BCC), its classification still remains controversial. FEP may also be categorized as a variant of trichoblastoma, a benign counterpart of BCC [2].

Typically, the patients are male, 40–60 years of age and present with a single or multiple pedunculated or sessile nodules with broad base on the trunk or extremities [3]. The tumor may appear pink, red, brown or skin color with occasional ulceration. Histologically, the tumor is composed of long, thin, and branching strands of basal cell carcinoma anastomosing in fibrovascular stroma [3].

The primary differential diagnoses of FEP include BCC, trichoblastoma, and trichoepithelioma. The histologic appearance of the tumor is usually distinctive. Androgen receptor is expressed both in BCC and FEP but minimally in trichoblastomas [4]. On the other hand, Merkel cells are found in both FEP and trichoblastoma but they are absent in BCC [2].

The pathogenesis of FEP is still investigational. It is thought that a mutation in the tumor suppressor gene *TP53 *might predispose to the development of FEP [5]. Similarly to BCC, the suggestion has been made that mutations in the *PATCHED *gene, which provides inhibitory signal in the Hedgehog pathway, could also lead to the development of FEP [2,3,5]. Further studies are needed to further elucidate the genetic predisposition of FEP.

The definitive treatment for FEP is complete excision. The tumor is considered an indolent basal cell carcinoma with no metastatic potential. Other treatment options include electrodessication and curettage, cryosurgery, or Moh's micrographic surgery [2,3].

## Consent

Written consent was obtained from the parents of the patient for publication of this case report. A copy of the written consent is available for review by the Editor-in-Chief of this journal.

## Competing interests

The authors declare that they have no competing interests.

## Authors' contributions

ZP reviewed the literature and prepared the photomicrograph, NH drafted the manuscript, and DPS conceived, revised, and submitted the manuscript. All authors have read and approved the final manuscript.
